# Evolution of nucleic acid amplification testing across Canada as observed through the Canadian Laboratory Response Network’s SARS-CoV-2 Proficiency Test Program, May 2020 to June 2021

**DOI:** 10.14745/ccdr.v49i05a02

**Published:** 2023-05-01

**Authors:** Charlene Ranadheera, Kym Antonation, Cindi Corbett

**Affiliations:** 1 Health Security and Response Division, National Microbiology Laboratory, Public Health Agency of Canada, Winnipeg, MB

**Keywords:** proficiency test scheme, SARS-CoV-2, molecular testing, COVID-19

## Abstract

To help accommodate the surge in severe acute respiratory syndrome coronavirus 2 (SARS-CoV-2) clinical testing due to the coronavirus disease 2019 pandemic, the decentralization of testing from provincial public health laboratories to regional laboratories and private facilities was necessary. To further support the growing number of test sites in Canada, the National Microbiology Laboratory developed a proficiency test program for the detection of SARS-CoV-2 using nucleic acid amplification tests and administered it under an arm of the Canadian Laboratory Response Network (CLRN). Since its conception in May 2020, CLRN has conducted three proficiency test schemes, from May 2020 to June 2021, and has observed an increase in participation of more than 400%. This article will explore the evolution of CLRN’s SARS-CoV-2 Proficiency Test Program and its support of the Canadian pandemic response.

## Introduction

In December 2019, a virus capable of causing acute respiratory disease in humans was reported in the Wuhan area, within the Hubei province of China. Since then, this virus, severe acute respiratory syndrome coronavirus 2 (SARS-CoV-2) has spread rapidly around the world and has led to the most significant global pandemic of the 21^st^ century. Rapid identification and contact tracing are essential to maintaining and managing critical public health infrastructure. Due to the ever-growing number of coronavirus disease 2019 (COVID-19) cases, it was necessary to establish decentralized testing and equipment laboratories, hospitals and healthcare centres with the ability to conduct SARS-CoV-2 diagnostics independently. Nucleic acid amplification tests (NAAT) are the current standard for diagnosis. In addition to equipping and training these centres for testing, accreditation and licensing to conduct SARS-CoV-2 testing were required.

In April 2020, a request for support from the Canadian Public Health Laboratory Network was made to the Canadian Laboratory Response Network (CLRN) for the rapid provision of a SARS-CoV-2 Proficiency Test Program to aid the provincial and regional public health partners, since commercial proficiency test programs were not available at the time. The first CLRN SARS-CoV-2 proficiency test scheme was distributed through the National Microbiology Laboratory in May 2020, a second one in November 2020 and a final one in June 2021. Since then, a number of national and international organizations have developed open-participation proficiency test programs for SARS-CoV-2, allowing for de-escalation of this national emergency support measure. Concurrent with the CLRN testing program, in March 2021, the Canadian Microbiology Proficiency Testing organization deployed their first SARS-CoV-2 proficiency test scheme, consisting of four test samples, simulating fresh swab specimens with three shipments per year ((1)). The College of American Pathologists distributed their first SARS-CoV-2 molecular test scheme in November 2021, consisting of three liquid simulated respiratory specimens, with two shipments per year ((2)). Quality Control for Molecular Diagnostics, another international external quality assurance provider, delivers a five-specimen SARS-CoV-2 panel annually ((3)). The World Health Organization also hosts an external quality assurance program for SARS-CoV-2; however, this program is limited to national and subnational laboratories around the world ((4)).

This article discusses the various trends and insights into SARS-CoV-2 testing observed in Canada from May 2020 to June 2021 through the delivery of the CLRN SARS-CoV-2 Proficiency Test Program.

## Results and discussion

**Figure 1:**
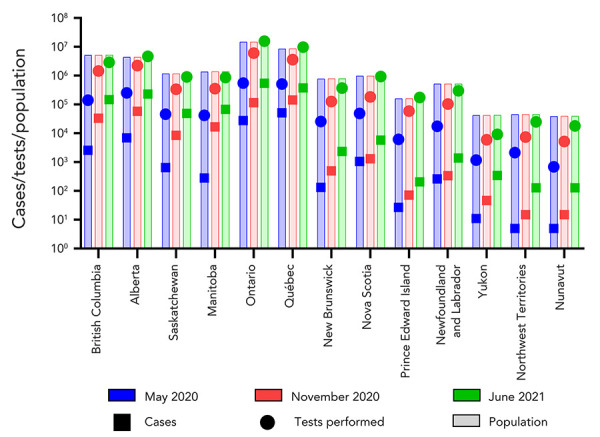
Population and SARS-CoV-2 demographics across Canada^a^ Abbreviation: SARS-CoV-2, severe acute respiratory syndrome coronavirus 2 ^a^ The number of SARS-CoV-2 cases, tests performed, and case counts across the country were tracked using data collected from the Government of Canada’s Coronavirus disease (COVID-19): Outbreak update reference page ((5)). Population counts at each time point were determined using the Government of Canada’s population estimates tool ((6)). A breakdown of cumulative COVID-19 cases per province is presented (square). A breakdown of cumulative COVID-19 tests performed per province are presented (circle). An estimated of provincial/territorial population is presented (faded bar)

**Figure 2:**
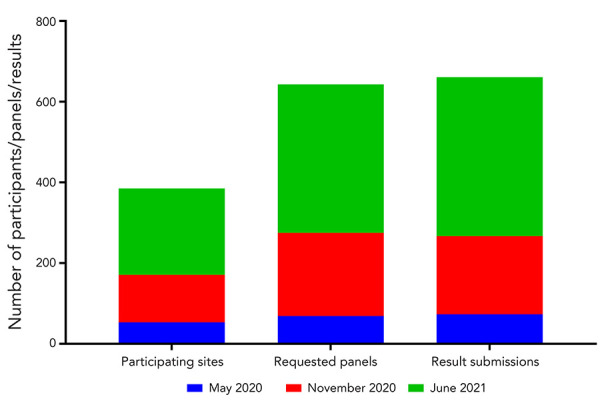
The number of participating sites, requested panels and results submitted over the course of the three CLRN SARS-CoV-2 proficiency test schemes Abbreviations: CLRN, Canadian Laboratory Response Network; SARS-CoV-2, severe acute respiratory syndrome coronavirus 2

**Figure 3:**
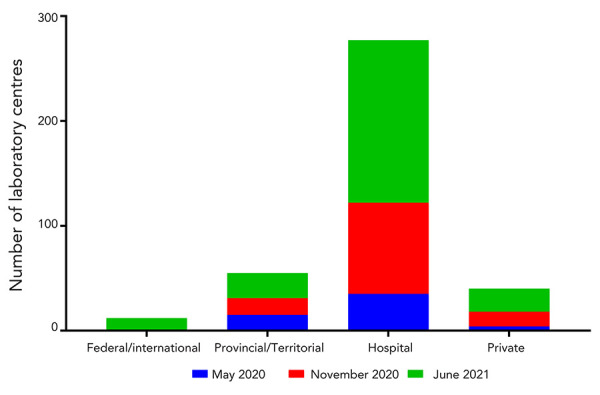
The number of jurisdictional laboratory centres participating in CLRN’s SARS-CoV-2 Proficiency Test Program period Abbreviations: CLRN, Canadian Laboratory Response Network; SARS-CoV-2, severe acute respiratory syndrome coronavirus 2

Three CLRN proficiency test schemes, which make up the CLRN Proficiency Test Program, were distributed to public health partners between May 2020 and June 2021. Participants were provided with six contrived-clinical test samples containing inactivated virus and were asked to employ their respective laboratory’s algorithms for NAAT to detect the presence of SARS-CoV-2. As the pandemic evolved, testing demands and COVID-19 cases across the country increased dramatically (**Figure 1**). As such, it was necessary to further decentralize testing and expand testing centres to include regional hospitals and private laboratories. By June 2021, significant scale up by every province and territory was evident, including the increase in testing capacity in northern, remote and isolated communities, which normally would have depended on large urban facilities (Figure 1) ((5,6)). This trend was reflected over the course of three CLRN proficiency test schemes. The CLRN engaged with provincial and territorial jurisdictional partners to identify appropriate participants. Fifty-three laboratories participated in the May 2020 test scheme and participants increased to 118 and 214 for the November 2020 and June 2021 test schemes, respectively (**Figure 2**). Increasing participation at all Canadian jurisdictional levels from May 2020 to June 2021 was observed; provincial laboratories increased site participation by 160% (nine new participating centres), regional hospital participation grew by 443% (120 new participating centres) and private laboratories expanded by 550% (18 new participating centres) (**Figure 3**).

Furthermore, we observed a 285% increase, between May 2020 to June 2021, in laboratory participation from partners located in remote and isolated communities in northern Canada. Canadian Federal Surge Laboratories, sites that support the overflow of public health samples from provincial laboratories, participated for the first time and accounted for seven new centres during the June 2021 test scheme (Figure 3). Finally, members of the Global Health Security Action Group, involving five international participants, participated in the June 2021 test scheme.

Increased testing nationwide correlated with an increase in test panel requests and result submissions: from 69 and 73 for the May 2020 test scheme, respectively; to 206 and 194 for the November 2020 test scheme, respectively; and 368 and 394 for the June 2021 test scheme, respectively (Figure 2). Additional breakdown by geographical or population demographics was not possible since collection of secondary metadata was not done and variations between jurisdictional participation due to resource limitations at the time would misrepresent any observations that could be made. As participation in this proficiency test program was successfully embraced by all partners, there were logistical challenges surrounding the facilitating of the large-scale test distribution in a short period of time. Future planning needs to be mindful of inter-provincial networks, available resources and rapid deployment of material transfer agreements.

**Figure 4:**
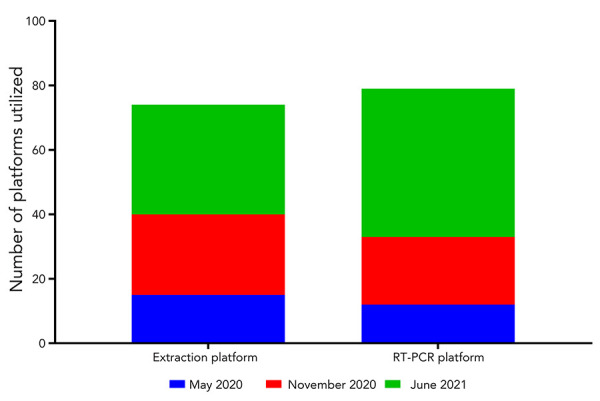
The number of different extraction and real-time PCR platforms used in each CLRN’s SARS-CoV-2 proficiency test scheme Abbreviations: CLRN, Canadian Laboratory Response Network; RT-PCR, reverse transcription polymerase chain reaction; SARS-CoV-2, severe acute respiratory syndrome coronavirus 2

The variety of nucleic acid extraction and reverse transcription polymerase chain reaction (RT-PCR) platforms expanded and correlated with the surge in countrywide testing; there was a 227% and 383% rise in different extraction and RT-PCR platforms used, respectively (**Figure 4**).

The May 2020 proficiency test scheme had 53 participants submitting 73 sets of panel results (all results were as expected), while the November 2020 test scheme had 118 participants submitting 194 sets of results (94.3% obtaining expected results) and the June 2021 test scheme had 214 participants submitting 394 sets of results (99.5% obtaining expected results). Consistent with the high success rates, results were comparable across provincial, regional and private facilities, with no discernable pattern associated with discordant results. The only exception was seen during the November 2020 test scheme, where a marginally lower success rate was observed in comparison to the other two test schemes. These results correlated with the inclusion of regional facilities to support an increase in testing capacity. Result discrepancies were identified and corrective actions were proposed through the evaluation portion of the test program. Successful remediation and functional workflows were observed in the subsequent June 2021 test scheme.

As capacity grew across Canada and as the pandemic approached the 2020 “cold and flu” season, the need for laboratories to distinguish between SARS-CoV-2 and other respiratory pathogens of significance grew. Many testing facilities began running multiplexed RT-PCR assays or equivalent assays to test for a multitude of respiratory pathogens, including SARS-CoV-2. To support this, participants had the option to report on other respiratory pathogens that may have been detected during their testing. The November 2020 test scheme was modified and made up of six contrived-clinical samples: three samples containing SARS-CoV-2, one sample containing both SARS-CoV-2 and respiratory syncytial virus (RSV), one sample containing influenza A virus, and one sample with no virus. Twenty-four participants implemented testing parameters to detect influenza A virus and 22 implemented testing for RSV; all participants correctly identified the samples containing these viruses. The June 2021 test scheme extended these parameters to consist of two contrived-clinical samples containing varying amounts of SARS-CoV-2, two contrived clinical samples containing SARS-CoV-2 and rhinovirus or influenza B virus, one contrived clinical sample containing influenza A virus and one containing no virus. Fifty-four sites employed rhinovirus testing, 116 sites implemented testing parameters for influenza A virus and 106 sites conducted influenza B virus testing; in all cases the various viruses were correctly identified in their respective samples. Only one discordant result was observed, an equivocal RSV result was obtained for a sample containing SARS-CoV-2 only.

The emergence of SARS-CoV-2 variants of concern (VOCs) became a reality in the latter part of 2020. The first SARS-CoV-2 VOC (B.1.1.7) is suspected to have emerged in the United Kingdom, with the earliest samples reported in September 2020, and had spread to multiple countries by December 2020 ((7,8)). Ontario confirmed Canada’s first case of the B.1.1.7 variant on December 26, 2020, and by April 26, 2021, all provinces and territories had reported confirmed cases. Since then, VOCs continued to emerge and spread throughout the world ((8)) and laboratories and reference facilities began developing assays to identify and flag VOCs. To further support these laboratories, the June 2021 CLRN SARS-CoV-2 proficiency test scheme incorporated three SARS-CoV-2 VOCs into the test panel. The June 2021 test scheme had samples containing the SARS-CoV-2 wild-type virus, B.1.1.7, B.1.351 or P.1 variants. Forty-seven participants performed a variety of short nucleotide polymorphism (SNP) assays and two participants conducted whole genome sequencing. Sixty-eight percent of participants identified the sample containing the B.1.1.7 variant, while 24% reported detection of an unspecified variant and 8% were incorrect or undetermined. Thirty-nine percent of participants identified the sample containing the B.1.351 variant, while 59% identified either B.1.351/P.1 variants, and 2% reported detection of an unspecified variant. Twelve percent of participants correctly identified the sample containing the P.1 variant, while 51% identified either B.1.35/P.1, 22% reported detection of an unspecified variant, and 14% were incorrect or undetermined. Finally, 78% of participants correctly identified the wild-type strain while 22% were undetermined. Overall, the majority of sites were able to identify the presence of a VOC; however, typing the variant utilizing SNP assays was inconsistent due to a limited combination of assays being used and would require additional SNP assays or genomic sequence analysis to get a definitive lineage. For example, the B.1.351 and P.1 variants both share an E484K and N501Y mutation in the spike protein; without a distinguishing target, such as K417N/T, identifying a lineage would not be possible.

Therefore, understanding the objective and subsequent public health outcome is necessary to determine the complexity of the workflows required. While whole genome sequencing provides a large dataset, there are a number of advantages to using SNP assays: higher throughput; increased sensitivity; reduced impact on resources and infrastructure; and better cost effectiveness.

## Conclusion

The provision of the CLRN SARS-CoV-2 Test Program from May 2020 to June 2021 demonstrated the scaleability of Canadian public health external quality assurance programs through the CLRN. Having a centralized Canadian proficiency test program enabled the laboratory network to identify performance metrics and considerations, such as the need to expand testing assays for VOC identification, if laboratories prefer to discriminate between circulating VOCs with a PCR screen. The comprehensive program also demonstrated the fluidity of the public health system in Canada to adapt to rapidly changing environments. A hallmark of the Canadian laboratory response to the COVID-19 pandemic was the rapid and successful implementation of testing laboratories across the country to accommodate the surge in testing requirements. Whether it was increasing testing capacity in urban settings through the participation of hospital laboratories, private facilities and federal surge sites, or implementing testing centres in remote and isolated communities in northern Canada, the CLRN SARS-CoV-2 Proficiency Test Program clearly demonstrated successful surge capacity while maintaining testing standards, providing Canadians with rapid identification of SARS-CoV-2 infection.
